# Relationship between one‐carbon metabolism and fetal growth in twins: A cohort study

**DOI:** 10.1002/fsn3.3611

**Published:** 2023-08-11

**Authors:** Xiaoli Gong, Xiaona Li, Yufeng Du, Jing Yang, Xuening Li, Jiaxin Li, Yangyu Zhao, Yuan Wei

**Affiliations:** ^1^ Department of Obstetrics and Gynecology, National Clinical Research Center for Obstetrics and Gynecology, National Center for Healthcare Quality Management in Obstetrics Peking University Third Hospital Beijing China; ^2^ Department of Pharmacy Peking University Third Hospital Beijing China; ^3^ Department of Epidemiology and Statistics, School of Public Health Lanzhou University Lanzhou China

**Keywords:** fetal growth, one‐carbon metabolism, twins cohort study

## Abstract

We investigated the associations of one‐carbon metabolism (OCM)‐related metabolites, including choline, betaine, dimethylglycine (DMG), and methionine with fetal growth of twins. This hospital‐based cohort study included dichorionic twin gestations. Blood samples were collected at a median of 14.7 weeks of gestation. Blood plasma metabolite levels were measured using high‐performance liquid chromatography–triple quadrupole mass spectrometry. Generalized estimating equations and mixed effects models were used to explore associations between plasma metabolite levels and fetal growth. In total, 115 women with dichorionic diamniotic pregnancies were included. The maternal plasma DMG level was negatively correlated with fetal birth weight (*β* = −43.5, 95% confidence interval [CI] = −74.1 to −12.8, *p* < .05) and head circumference (*β* = −0.23, 95% CI = −0.39 to −0.07, *p* < .05). Other metabolites were not significantly associated with birth weight, body length, head circumference (HC), or chest circumference. Analysis of the relationships between plasma metabolite levels and fetal biological parameters on ultrasound revealed that the maternal choline level was negatively correlated with fetal abdominal circumference (AC) (*β* = −0.12, 95% CI = 0.24 to −0.004, *p* < .05); the maternal DMG level was negatively correlated with fetal AC (*β* = −0.17, 95% CI = 0.28–0.07, *p* < .05), femur length (*β* = 0.02, 95% CI = 0.04–0.003, *p* < .05), and estimated fetal weight (*β* = 26.4, 95% CI = −41.6 to −11.2, *p* < .05), but not with HC. The maternal methionine level was negatively correlated with HC (*β* = −0.08, 95% CI = −0.14 to −0.02, *p* < .05). The plasma level of the OCM‐related metabolite DMG during the second trimester was negatively correlated with fetal intrauterine growth and birth weight. However, further studies with larger samples are needed.

## Graphical abstract

We investigated the associations of one‐carbon metabolism‐related metabolites, including choline, betaine, dimethylglycine (DMG), and methionine with fetal growth of twins. The plasma level of the DMG during the second trimester was negatively correlated with fetal intrauterine growth and birth weight.

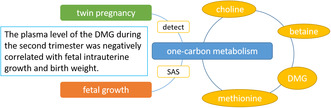

## 1 INTRODUCTION

Developmental Origins of Health and Disease (DOHaD) is a new theory regarding the origin of human diseases. According to the DOHaD theory, in addition to lifestyle and genetic factors, environmental and nutritional factors in early human life affect the prevalence of specific adult diseases (e.g., obesity, diabetes, and cardiovascular diseases; Bianco‐Miotto et al., 2017). The developing fetus relies on nutrients provided by the mother to maintain rapid cell division. Damage during this critical period may result in permanent metabolic or structural changes, leading to diseases in adulthood. The DOHaD theory is based on epigenetic mechanisms, which produce heritable changes in gene expression that do not involve changes in DNA sequences. Epigenetic mechanisms regulate gene expression through DNA methylation, histone modification, chromatin remodeling, and noncoding RNA. Methylation is a key method for the modification of proteins and nucleic acids, with central roles in multiple diseases (Cao‐Lei et al., 2020). Methylation requires methyl donors, and mammals rely on one‐carbon metabolism (OCM) to provide methyl groups for methylation reactions. Furthermore, OCM relies on methyl donors from food sources, such as choline, folate, methionine, and betaine (Deng & Wang, 2021; Mentch & Locasale, 2016).

Choline is an essential nutrient for cell membrane integrity, cholinergic neurotransmission, and OCM (Zeisel, 2006). In OCM, choline is irreversibly oxidized to betaine, which provides a methyl group to homocysteine (Hcy) to form methionine through the action of betaine homocysteine methyltransferase; this methyl supply pathway functions in a manner similar to folic acid metabolism. Methionine metabolism, folic acid metabolism, and sulfur transfer are the three basic types of OCM in the human body. During sulfur transfer, serine and Hcy are catalyzed by enzymes to synthesize cystathionine. OCM is involved in various important physiological responses, life stages, and diseases, such as congenital dysplasia (Raad & AbuAlhommos, 2021), nonalcoholic fatty liver disease (Corbin & Zeisel, 2012), cancer (Lauinger & Kaiser, 2021), vascular disease (Tian et al., 2020), and dementia. There is evidence that OCM‐related metabolites (e.g., choline, betaine, and methionine) in the maternal blood (van Lee et al., 2016), amniotic fluid (Monsen et al., 2006), and umbilical cord blood (Hogeveen et al., 2013; Ivorra et al., 2012) are positively or negatively correlated with fetal birth weight or body length in singleton pregnancies. However, few studies have evaluated the associations of OCM‐related metabolites in twin pregnancies.

Based on previous literature, we speculated that OCM‐related metabolites, including choline, betaine, dimethylglycine (DMG), and methionine, have important roles in fetal growth. We evaluated this relationship in a cohort of twin pregnancies using targeted metabolomics analysis.

## 2 METHODS

### 2.1 Study design

This twin pregnancy cohort study recruited pregnant women who underwent regular follow‐up at the Peking University Third Hospital between November 2018 and November 2019. The study included women who had dichorionic diamniotic (DCDA) pregnancies with live births of both twins. We excluded cases with severe maternal complications, pregnancy‐related complications (e.g., hemolysis, elevated liver enzymes, low platelets syndrome, severe preeclampsia, or other acute or chronic diseases), fetal chromosomal abnormalities, and/or congenital malformation.

We used medical records to collect demographic information (e.g., age, height, prepregnancy weight, nationality, and education level), as well as clinical data, including previous parity, disease history, mode of pregnancy, complications, gestational age, mode of delivery, and presence of premature rupture of membranes. Gestational age was calculated according to the last menstruation date and confirmed by ultrasound during early pregnancy; in cases with difference >7 days, the gestational age determined by ultrasound was used. In cases of conception through assisted reproductive technology, the last menstruation date was calculated by subtracting 17 or 19 days from the date of transfer, depending on the type of embryo transferred. The following fetal data were collected: ultrasound data (estimated fetal weight, biparietal diameter [BPD], head circumference [HC], femur length [FL], and abdominal circumference [AC] of twins), sex, birth weight and length, and chest circumference after birth.

### 2.2 Sample collection and preparation

Fasting blood of pregnant women were collected using ethylenediaminetetraacetic acid anticoagulant tubes during the first or early second trimester of pregnancy. After centrifugation, the upper layer of plasma was divided into 1.5‐mL Eppendorf tube and stored at −80°C until further analysis.

Sample preparation was conducted according to our previous study (Gong et al., 2021). Briefly, 50 μL of plasma was added into 30 μL of 0.1 M dithiothreitol, vortexed for 5 min, and then incubated for 10 min at room temperature. The mixtures were followed by the addition of 300 μL of methanol containing the internal standards (choline‐d9, betaine‐d3, DMG‐d6, and methionine‐d3), then centrifuged at 15,000 g for 15 min at 4°C. The supernatant was analyzed using high‐performance liquid chromatography–mass spectrometry.

### 2.3 Measurement of plasma metabolite level

We used ultra‐high‐performance liquid chromatography (UHPLC) coupled with triple quadrupole mass spectrometry (Triple Quad 4000 MS; AB Sciex) to analyze plasma levels of choline, betaine, DMG, and methionine. An XBridge BEH Amide column (1.7 μm, 2.1 × 100 mm; Waters) was used for metabolite separation. Sample injection volume was 1 μL, and column temperature was 35°C. Mobile phases A and B were acetonitrile with 0.1% formic acid and water with 0.1% formic acid and 5 mM NH_4_OMe, respectively. The gradient program was initiated at 15% B for 0.01–0.2 min, slowly increased to 45% B at 8 min, increased to 80% B at 8.5 min, maintained for 2 min, then decreased to 15% at 11 min, and maintained for 4 min. The targeted metabolites and their internal standards were monitored using positive ions in the multiple reaction monitoring (MRM) mode. Mass spectrometry parameters were optimized for each metabolite and internal standard. The concentration of each analyte was calculated using calibration curve with weighted least‐square (1/X2) linear regression algorithm. The interday coefficients of variation of quality controls at low, medium, and high concentrations were not more than 8.0%. All samples were blinded and measured by liquid chromatography/mass spectrometry technician.

### 2.4 Statistical analyses

Metabolite levels were presented as medians with interquartile ranges since the concentrations of all biomarkers were nonnormal distributions. The Mann–Whitney *U*‐test or Kruskal–Wallis test was used to compare metabolite levels across groups when baseline characteristics were compared. Confounding factors were gradually controlled by three models: Model 1: Correction for gestational age and fetal sex; Model 2: Covariables in Model 1 were added age of pregnant women, prepregnancy BMI, gestational age of blood collection, birth time, mode of conception, hypertensive diseases during pregnancy, and GDM; Model 3: Covariates in Model 2 are corrected with five metabolites. No pregnant women in this study were drinking or smoking, and they were all married, 92.2% of the nation were Han, so these variables were not corrected. SPSS (version 23.0; IBM Corp.) and R (version 3.6.1; R Foundation for Statistical Computing) software were used for statistical analyses. Data were subjected to logarithmic transformation. Differences with *p <* .05 were considered statistically significant. The relationships of metabolite levels with fetal birth weight, body length, HC, and chest circumference were analyzed using generalized estimating equations; the relationships of metabolite levels with biometric parameters on ultrasound were analyzed using mixed effects models.

## 3 RESULTS

This study included 115 women with DCDA pregnancies (median gestational age: 14.7 weeks [interquartile range: 12–17 weeks]). Choline levels were higher in primiparous women and in women who underwent assisted reproduction than in multiparous women or women with unassisted conception. Stratified analyses of the other characteristics, such as age and body mass index, revealed no significant differences according to metabolite levels (Table 1).

**TABLE 1:** Stratified analysis of the concentration of metabolites in maternal blood and the general characteristics of maternal and fetus^a^.

	*n* (%)	Choline	Betaine	DMG	Methionine
Age					
<35	74 (64.3)	13.0 (10.7, 15.7)	15.2 (12.8, 18.9)	1.7 (1.3, 1.9)	14.5 (12.7, 17.1)
≥35	41 (35.7)	12.7 (11.0, 14.7)	14.9 (12.2, 17.5)	1.6 (1.3, 2.0)	13.7 (11.2, 16.2)
*p*		.76	.42	.90	.12
Nationality					
Ethnic Han	106 (92.2)	12.7 (10.8, 14.7)	14.9 (12.6, 17.5)	1.6 (1.3, 1.9)	14.1 (12.1, 17.0)
National minority	9 (7.8)	14.4 (11.0, 15.9)	18.2 (12.6, 20.9)	1.8 (1.1, 2.1)	15.7 (10.9, 17.5)
*p*		.39	.22	.80	.77
Prepregnancy BMI					
<18.5	8 (7.0)	13.1 (10.1, 15.6)	16.1 (13.2, 20.4)	1.8 (1.5, 2.0)	13.5 (11.3, 16.7)
18.5–24.9	71 (61.7)	12.2 (10.0, 14.4)	15.3 (12.7, 19.2)	1.7 (1.2, 2.0)	14.1 (12.1, 17.1)
≥25	36 (31.3)	13.3 (11.9, 16.2)	13.8 (12.3, 16.7)	1.5 (1.3, 1.8)	14.4 (12.1, 16.9)
*p*		.08	.10	.44	.89
Parity					
Primipara	106 (92.2)	13.0 (11.4, 15.0)	15.2 (12.7, 17.8)	1.7 (1.3, 2.0)	14.2 (12.3, 17.0)
Multipara	9 (7.8)	9.4 (8.0, 12.7)	11.7 (9.6, 16.5)	1.1 (1.0, 1.9)	14.0 (10.8, 18.2)
*p*		.01*	.10	.08	.86
Mode of conception					
Nature	15 (13.0)	11.4 (8.4, 13.4)	15.5 (10.9, 17.5)	1.3 (1.0, 2.2)	15.0 (12.7, 18.4)
ART	100 (87.0)	13.0 (11.4, 15.5)	15.0 (12.7, 18.1)	1.7 (1.3, 2.0)	14.1 (12.0, 16.9)
*p*		.01*	.58	.14	.28
Gestational age of sample					
<14.7	57 (49.6)	12.2 (10.3, 14.7)	15.5 (12.8, 18.5)	1.6 (1.2, 1.9)	14.0 (11.8, 17.1)
≥14.7	58 (50.4)	13.1 (11.3, 15.6)	14.1 (12.5, 17.6)	1.7 (1.3, 2.0)	14.2 (12.6, 17.0)
*p*		.24	.14	.23	.81
Fetal sex					
Boy–boy	38 (33.0)	12.2 (10.6, 14.9)	14.0 (12.2, 17.6)	1.5 (1.2, 1.8)	13.9 (12.1, 16.6)
Boy–girl	31 (27.0)	13.4 (11.6, 16.2)	15.2 (13.2, 19.5)	1.8 (1.3, 2.1)	14.3 (12.3, 16.7)
Girl–girl	46 (40.0)	13.0 (10.8, 14.4)	15.4 (12.8, 17.3)	1.7 (1.3, 1.9)	14.6 (12.3, 17.4)
*p*		.35	.54	.22	.66

^a^ 25th‐Mann–Whitney *U*‐test and Kruskal–Wallis test were used to calculate the *p* values in n (%), the median (75th) of metabolite concentrations.

* *p* < .05.

Tables 2, 3, 4, 5 present the relationships of metabolite levels with fetal outcomes. The maternal plasma DMG level was negatively correlated with fetal birth weight (*β* = −43.5, 95% confidence interval [CI] = −74.1 to −12.8, *p* < .05) and HC (*β* = −0.23, 95% CI = −0.39 to −0.07, *p* < .05). No other metabolite levels were significantly associated with birth weight, body length, HC, or chest circumference.

**TABLE 2:** Relationship between metabolite concentration and birth weight of newborn.

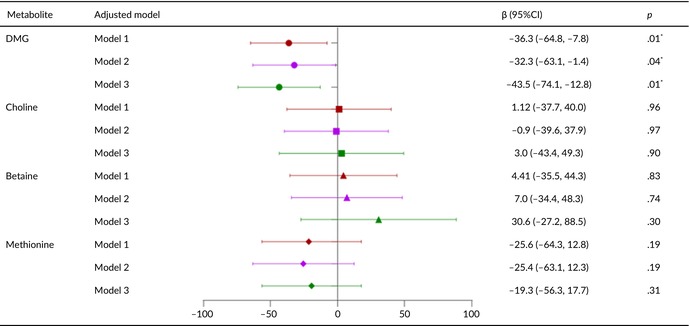

*Note*: Model 1: Correction for gestational age and fetal sex; Model 2: Covariables in Model 1 were added age of pregnant women, prepregnancy BMI, gestational age of blood collection, birth time, mode of conception, hypertensive diseases during pregnancy, and GDM; Model 3: The covariable in Model 2 is corrected by adding five metabolites.

* *p* < .05.

**TABLE 3:** Relationship between metabolite concentration and length of newborn.

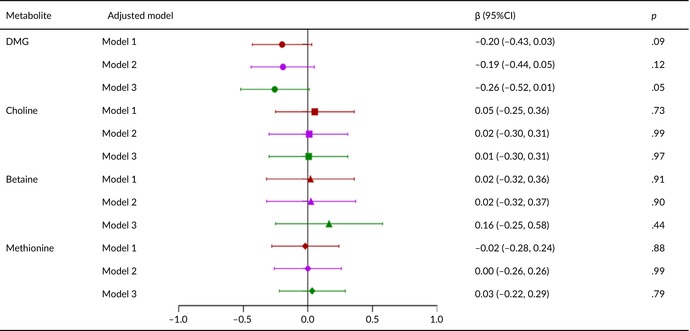

*Note*: Model 1: Correction for gestational age and fetal sex; Model 2: Covariables in Model 1 were added age of pregnant women, prepregnancy BMI, gestational age of blood collection, birth time, mode of conception, hypertensive diseases during pregnancy, and GDM; Model 3: Covariates in Model 2 are corrected with five metabolites.

**TABLE 4:** Relationship between metabolite concentration and head circumference of newborn.

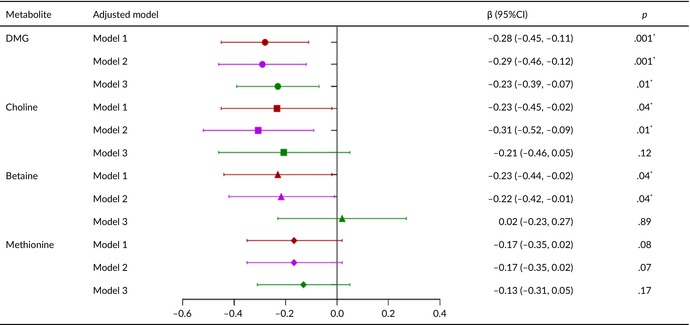

*Note*: Model 1: Correction for gestational age and fetal sex; Model 2: Covariables in Model 1 were added age of pregnant women, prepregnancy BMI, gestational age of blood collection, parity, mode of conception, hypertensive diseases during pregnancy, and GDM; Model 3: Covariates in Model 2 are corrected with five metabolites. *p < .05.

**TABLE 5:** Relationship between metabolite concentration and chest circumference of newborn.

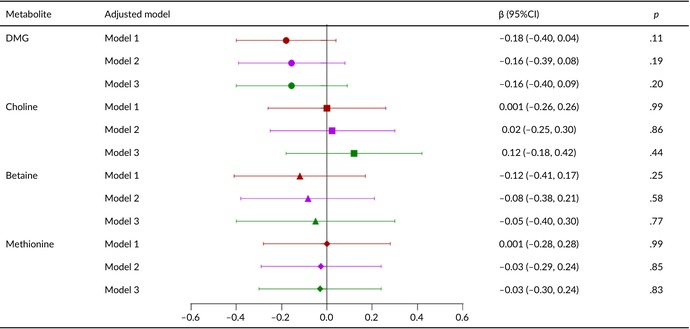

*Note*: Model 1: Correction for gestational age and fetal sex; Model 2: Covariables in Model 1 were added age of pregnant women, prepregnancy BMI, gestational age of blood collection, parity, pregnancy mode, hypertensive diseases during pregnancy, and GDM; Model 3: Covariates in Model 2 are corrected with five metabolites.

Analyses of the relationships of metabolite levels with fetal biological parameters on ultrasound (Table 6) revealed that the maternal choline level was negatively correlated with fetal AC (*β* = −0.12, 95% CI = 0.24 to −0.004, *p* < .05), whereas the maternal DMG level was negatively correlated with fetal AC (*β* = −0.17, 95% CI = 0.28–0.07, *p* < .05), femur length (*β* = 0.02, 95% CI = 0.04–0.003, *p* < .05), and estimated fetal weight (*β* = 26.4, 95% CI = −41.6 to −11.2, *p* < .05), but not with HC. The maternal methionine level was negatively correlated with HC (*β* = −0.08, 95% CI = −0.14 to −0.02, *p* < .05).

**TABLE 6:** Relationship between metabolite concentration and ultrasonic biometric indicators.

	BPD		HC		AC		FL		EFW	
	*β* (95% CI)	*p*	*β* (95% CI)	*p*	*β* (95% CI)	*p*	*β* (95% CI)	*p*	*β* (95% CI)	*p*
Choline	0.02 (−0.01, 0.05)	.13	−0.05 (−0.14, 0.03)	.22	−0.12 (−0.24, −0.004)	.04*	−0.02 (−0.04, 0.00)	.05	−13.6 (−30.3, 3.09)	.11
Betaine	−0.01 (−0.04, 0.02)	.36	0.01 (−0.08, 0.10)	.82	0.07 (−0.04, 0.19)	.23	0.01 (−0.02, 0.03)	.60	8.79 (−8.17, 25.8)	.31
Dmg	0.003 (−0.02, 0.03)	.84	−0.01 (−0.09, 0.07)	.79	−0.17 (−0.28, −0.07)	.001*	−0.02 (−0.04, −0.003)	.02*	−26.4 (−41.6, −11.2)	<.001*
Methionine	−0.002 (−0.02, 0.02)	.85	−0.08 (−0.14, −0.02)	.01*	−0.05 (−0.14, 0.03)	.23	0.003 (−0.01, 0.02)	.70	−3.47 (−16.0, 9.06)	.58

*Note*: Correction for gestational age, prepregnancy BMI, gestational age of blood collection, fetal sex, parity, mode of conception, GDM, hypertensive disease during pregnancy, and five metabolites.

* *p* < .05.

## 4 DISCUSSION

OCM is involved in multiple important physiological reactions in the human body; it is closely associated with fetal growth and development. Here, we used targeted metabolomics to investigate the relationships of OCM‐related metabolites in DCDA pregnancies with fetal intrauterine growth, birth weight, and other parameters. The median gestational age among the study participants was 14.7 weeks. The maternal plasma DMG level was negatively correlated with fetal birth weight, HC at birth, AC, FL, and EFW measured on ultrasound during pregnancy; it was not correlated with HC on ultrasound. The maternal choline level was negatively correlated with AC on ultrasound, whereas the maternal methionine level was negatively correlated with HC on ultrasound.

Betaine provides a methyl group to Hcy through the activity of betaine homocysteine methyltransferase to produce DMG, which is the only pathway for endogenous synthesis of DMG. Therefore, the DMG level is also related to the activity of betaine homocysteine methyltransferase. DMG is demethylated through the activity of dimethylglycine dehydrogenase to produce sarcosine and glycine, which play central roles in various metabolic processes (e.g., synthesis of collagen, elastin, purine, creatine, and glutathione). Among these synthesis products, glutathione protects the body from oxidative stress; it also regulates the production of protein and cellular DNA. The removed methyl is transferred to 5,10‐methylene tetrahydrofolic acid, which enters the folate cycle and is converted to methyltetrahydrofolate via methylene tetrahydrofolic acid reductase; methyltetrahydrofolate then provides methyl to Hcy to produce methionine. This methyl‐supplying pathway functions in parallel to the betaine metabolic pathway; the two pathways complement each other, thus providing methyl for in vivo methylation reactions. During DMG metabolism, the in vivo methyl‐supplying pathway is connected to form a closed loop, which improves the rate of methyl utilization (Kou, 2016; Wang et al., 2018). The levels of betaine and DMG are lower, whereas the level of choline is higher, in women with normal pregnancy than in nonpregnant women. The high level of leukocyte choline dehydrogenase mRNA in pregnant women suggests that the decreased levels of betaine and DMG are caused by increased metabolism, rather than decreased synthesis (Friesen et al., 2007). Pregnant women have a high metabolic rate. High levels of choline, betaine, and DMG in umbilical cord blood suggest a high demand for methyl donors for fetal and placental growth (Friesen et al., 2007; Molloy et al., 2005).

To our knowledge, few studies have investigated the relationship between DMG level and fetal growth. The present study showed that, after adjustment for several variables, the maternal blood DMG level in DCDA pregnancies was negatively correlated with fetal intrauterine size and birth weight. Hogeveen et al. (2013) showed that the cord blood DMG level was positively correlated with fetal birth weight. There was a strong correlation (*r* = .58) between the DMG level in maternal blood and the DMG level in umbilical cord blood, confirming that the DMG in fetal circulation was obtained from maternal circulation. Additionally, a Brazilian study revealed high levels of DMG and methionine in breast milk during lactation (Hampel et al., 2020), as well as higher levels of urinary DMG, choline, betaine, and serine, in full‐term neonates fed breast milk compared with neonates fed formula milk (Shoji et al., 2020). Fetal demand for DMG suggests that it plays a key role in fetal growth. Animal experiments have shown that dietary supplementation with dimethylglycine sodium can effectively enhance liver antioxidant capacity and immunity in weaned piglets with fetal growth restriction (Feng et al., 2019); a similar effect has been observed in broilers (Kou et al., 2015). Fetal and placental oxidative stress may lead to fetal growth retardation. Glutathione is a nonenzymatic antioxidant, which can scavenge free radicals by inhibiting both free radicals and antisuperoxide anions. Low dietary choline levels can increase oxidative stress markers in rats, resulting in excessive accumulation of reactive oxygen species (Han et al., 2018). Choline supplementation can increase blood glutathione content and improve glutathione redox balance in children with cystic fibrosis who have low levels of choline (Bernhard, 2020). Glutathione content is low in the livers of piglets with fetal growth restriction; the activity of liver glutathione S‐transferase and the level of glutathione are increased after dietary supplementation with DMG (Feng et al., 2019). These findings indicate that glycine and glutathione have key roles in the normal placenta and fetus that involve the choline–betaine–DMG pathway, which scavenges free radicals to ensure normal fetal growth. In twin pregnancies, the mother and both fetuses require high levels of nutrients because of the increased placental area and high energy metabolism. Therefore, nutritional deficiency may occur in case of insufficient dietary intake. Additional studies are needed to determine whether women with twin pregnancies require additional supplementation of the aforementioned substances.

In the present study, targeted metabolomics was used to evaluate plasma levels of OCM‐related metabolites in women with DCDA pregnancies, and the results were compared with fetal growth outcomes. The maternal DMG level in the second trimester was negatively correlated with fetal intrauterine growth and birth weight. The strengths of this study were the accurate quantification of metabolite levels and the inclusion of adjustments for several confounding factors. Additionally, this study evaluated ultrasound parameters to explore relationships between metabolite levels and in utero fetal growth. Nevertheless, this study had some limitations. First, the Hcy level was not analyzed because of multiple outliers. Hcy is degraded within 2 h after sampling; therefore, a stabilizer should be added during sampling to ensure that subsequent test results are accurate. The lack of stabilizer in samples limited the accuracy of our analyses with respect to upstream and downstream metabolites of Hcy that are involved in fetal growth. Second, the sample size was small. Finally, the retrospective study design prevented the assessment of dietary intake during pregnancy. Therefore, the relationships between dietary intake and OCM metabolite levels could not be determined. Additional studies with larger sample sizes are needed to confirm our results and to elucidate specific regulatory mechanisms that underlie the effects of OCM on fetal growth.

## AUTHOR CONTRIBUTIONS

Yuan Wei and Yangyu Zhao were responsible for the overall study design and guided the research content. Xiaoli Gong, Xiaona Li, and Jiaxin Li collected data and samples. Yufeng Du and Xiaoli Gong performed the statistical analysis. Xuening Li, Xiaoli Gong, and Jing Yang conducted sample detection. Xiaoli Gong and Xiaona Li wrote the manuscript, and other co‐authors provided modification and improvement. All authors approved the final version of the manuscript. The corresponding authors had full access to the data in the study and takes responsibility for the integrity of the data.

## FUNDING INFORMATION

This study was supported by the National Natural Science Foundation of China (82171661), the National Key Research and Development Program of China (2021YFC2700700), and Peking University Third Hospital Cohort Building Program (BYSYDL2021004).

## CONFLICT OF INTEREST STATEMENT

The authors declare no conflict of interest.

## Data Availability

The data that support the findings of this study are available from the corresponding author upon reasonable request.
